# Long-Term Body Weight Maintenance among StrongWomen–Healthy Hearts Program Participants

**DOI:** 10.1155/2017/4372048

**Published:** 2017-03-02

**Authors:** Rebecca A. Seguin, Sara C. Folta, Miriam E. Nelson, Karla L. Hanson, Andrea Z. LaCroix

**Affiliations:** ^1^College of Human Ecology, Division of Nutritional Sciences, Cornell University, 412 Savage Hall, Ithaca, NY 14853, USA; ^2^Friedman School of Nutrition Science and Policy, Tufts University, 150 Harrison Avenue, Boston, MA 02111, USA; ^3^Sustainability Institute, University of New Hampshire, 107 Nesmith Hall, Durham, NH 03824, USA; ^4^College of Human Ecology, Division of Nutritional Sciences, Cornell University, 231 Savage Hall, Ithaca, NY 14853, USA; ^5^Family Medicine and Public Health, University of California, San Diego, 9500 Gilman Drive No. 0725, La Jolla, CA 92093, USA

## Abstract

*Background*. The repeated loss and regain of body weight, referred to as weight cycling, may be associated with negative health complications. Given today's obesity epidemic and related interventions to address obesity, it is increasingly important to understand contexts and factors associated with weight loss maintenance. This study examined BMI among individuals who had previously participated in a 12-week, evidence-based, nationally disseminated nutrition and physical activity program designed for overweight and obese middle-aged and older women.* Methods*. Data were collected using follow-up surveys. Complete height and weight data were available for baseline, 12-week program completion (post-program) and follow-up (approximately 3 years later) for 154 women (response rate = 27.5%; BMI characteristics did not differ between responders and nonresponders).* Results*. Mean BMI decreased significantly from baseline to post-program (−0.5, *P* < 0.001) and post-program to follow-up (−0.7, *P* < 0.001). Seventy-five percent of survey respondents maintained or decreased BMI post-program to follow-up. Self-efficacy and social support for healthy eating behaviors (but not physical activity) were associated with BMI maintenance or additional weight loss.* Conclusions*. These findings support the durability of weight loss following participation in a relatively short-term intervention.

## 1. Introduction

There is evidence that even modest amounts of weight loss can have beneficial effects on the health of obese individuals [1] and results can be achieved using a range of approaches, including programs that have focused on aerobic exercise [2], yoga [3], nutrition [4], combined diet and exercise [5], and mobile or electronic weight loss interventions [6].

It is important to note, however, that weight cycling—the repeated loss and regain of body weight—may be associated with health complications such as excess body weight, abdominal fat accumulation [7], type 2 diabetes [8], physical limitations [9], and negative impacts on immune function [10]. Arnold et al. [9] also reported a deleterious association of weight cycling with mortality. Some researchers examining methodological challenges in measuring the impact of weight cycling point to reverse causality in these analyses, while others maintain that while this may be true, weight cycling can be harmful for some populations [11]. Not surprisingly, those with a history of weight cycling are at risk for regaining weight [12]. However, a review of data on the prevalence of successful weight maintenance revealed that maintaining weight loss for at least 1 year is achievable by only approximately 20% of overweight individuals who lost 10% of their initial body weight [13].

Thus, it is important to understand the contexts in which weight loss is occurring among at-risk populations and to follow these individuals over time so that risk for weight loss regain can be addressed in intervention planning as well as future dissemination efforts [14]. Furthermore, the relationships between self-efficacy and social support and long-term maintenance of weight loss are poorly understood. This is due in part to a dearth of studies that examine longer-term outcomes [15]. In some studies, social support has been linked with greater effectiveness of interventions in promoting healthy eating, physical activity, and weight loss at 18 or more months [12, 15, 16]. However, in the Weight Loss Maintenance Trial [17] and in other studies [18], social support from family and friends appeared to hinder long-term maintenance of weight loss. Type of social support received may help explain these contradictory findings [18]. Associations between self-efficacy and long-term maintenance of weight loss have been somewhat more consistent [19–23].

This study was designed to examine the durability of body mass index (BMI; defined as weight in kilograms divided the square of height in meters) maintenance following participation in an evidence-based nationally disseminated program [the StrongWomen–Healthy Hearts Program (SWHH)] that targets cardiovascular disease risk reduction through dietary behavior change and regular aerobic exercise. The 12-week program demonstrated significant weight loss among overweight and obese women with a dose of hour-long twice weekly classes, in both the original community randomized trial (2007-2008) and the national dissemination (22 states) conducted between 2010 and 2013 [24]. Here we address the following research questions: (1) Among former participants in the SWHH program, how does BMI change from the time of program completion to follow-up (approximately three years later)? (2) What is the trajectory of change in BMI among women who maintained BMI versus those who increased BMI? and (3) Is self-efficacy and/or social support associated with follow-up BMI?

## 2. Materials and Methods

### 2.1. Data Collection, Participants, and Setting

From August 2013 to December 2013, 600 SWHH participants received invitations to participate in this study, the StrongWomen Follow-Up Study (SWFUS), in the form of an online or paper-based survey. A total of 165 participant responses were received (84 online and 81 on paper). Participants in this sample included women 40 years of age and older who lived independently, were sedentary, and had a BMI of 24 or higher at baseline [24, 25]. The analytic sample for this study included a total of 154 women (of the 165 respondents) for whom complete body weight data (height and weight) were available at all three points in time: baseline, post-program, and follow-up (approximately three years after baseline). The study was approved by the Institutional Review Board at Cornell University. Written consent was obtained from each participant.

### 2.2. Constructs and Measures

At baseline, height in centimeters (cm) was measured; weight was measured in kilograms at baseline and post-program. All measures were collected in triplicate using standard procedures [26] and those results have been previously published [24]. Follow-up height, in feet and inches, and body weight, in pounds, were self-reported by participants on the SWFUS survey. Height and weight were used to calculate BMI [27] for all points in time. For some analyses, post-program and follow-up BMI also were calculated as percentages of baseline BMI. Program BMI change was the difference between post-BMI and pre-BMI; follow-up BMI change was computed by follow-up BMI less post-program BMI. For some analyses, BMI changes also were calculated as percentages of baseline BMI. Baseline BMI is divided into three categories: overweight or at-risk of overweight (24.0–29.9), obese (30.0–34.9), and severely obese (≥35.0). Also, a dichotomous variable was computed for respondents as “yes, long-term BMI maintenance” if BMI decreased or remained unchanged from post-program to follow-up or “no long-term BMI maintenance” if BMI increased from post-program to follow-up.

Social factors were self-reported in the follow-up survey. Self-efficacy was measured by 16 items about self-efficacy for healthy eating and 14 items about self-efficacy for physical activity, adapted from Sallis et al. (1988) [28]. Scales for both self-efficacy for healthy eating (alpha = 0.92) and self-efficacy for physical activity (alpha = 0.95) ranged from 1 (not at all confident) to 5 (completely confident). Social support for healthy eating was measured by the validated Sallis Social Support Survey for Diet [29]. Four scales were created: family encouragement (alpha = 0.87), friend encouragement (alpha = 0.87), family discouragement (alpha = 0.82), and friend discouragement (alpha = 0.75), each of which ranges from 1 (never) to 5 (very often). Social support for physical activity was measured by the validated Sallis Social Support Survey for Exercise Behaviors [29]. Two scales were created: participation in physical activity among family (alpha = 0.88) and participation among friends (alpha = 0.93), each of which ranged from 1 (never) to 5 (very often). Baseline demographic data included age, race, marital status, education, and employment.

### 2.3. Statistical Analysis

Preliminary* t*-test and chi-square analyses were conducted to assess baseline demographic and anthropometric differences between SWHH respondents (with complete height and weight data for all three time points) and SWHH respondents without complete height and weight data for all three time points. The first analyses were conducted to assess change in BMI among SWHH Program participants, by comparing baseline BMI to post-program BMI and post-program BMI to follow-up BMI, using paired samples* t*-tests. The second analysis was conducted to test association between program BMI change and long-term maintenance of BMI (post-program to follow-up), using a chi-square test. The third analysis used one-way analysis of variance (ANOVA) to compare differences in program BMI change and follow-up BMI change among baseline weight status groups. In the final analyses, Pearson product-moment correlations and point-biserial correlations were run to determine the relationships among self-efficacy and social support for healthy eating and physical activity and “follow-up BMI change.”

Self-reported height is generally overreported, and body weight underreported [30] by approximately 1.89 kilograms (95% CI = 1.87, 1.91) among midlife and older women [31]. To adjust for these biases, we conducted two sensitivity analyses. First, we subsequently added the upper limit of this confidence interval to each respondent's follow-up body weight and recalculated BMI. Second, we also recalculated follow-up BMI using measured height from baseline. Mean “self-report adjusted” follow-up BMI, using these two methods, were compared to post-program BMI using a paired *t*-test, and results are provided in the text.

## 3. Results

### 3.1. Demographics

Baseline characteristics of follow-up survey SWHH respondents with complete height and weight data for all three time points (*n* = 154) and SWHH nonresponders (*n* = 446) were equivalent, except for the percentage married, which was higher among respondents than nonrespondents (77.7% versus 67.7%, resp., *P* = 0.03), Table 1. There was no difference between mean age at baseline among nonrespondents (mean = 60.1, ±SD = 10.0) and respondents (mean = 58.9, ±SD = 8.4), *P* = 0.19. The majority of the respondents and nonrespondents were white (*P* = 0.09) and had evenly distributed educational attainment across the categories of “high school or less,” “some college,” “bachelor's degree,” and “graduate training” (*P* = 0.81). There were no differences in the reported household income levels of respondents and nonrespondents, with both groups reporting household income levels less than $50,000 (*P* = 0.56). Importantly, baseline BMI was equivalent among nonrespondents and respondents (*P* = 0.74), as was BMI change from baseline to post-program among nonrespondents and respondents (*P* = 0.99).

### 3.2. Change in BMI

The results in Table 2 show mean BMI at baseline, post-program, and follow-up among participants. Mean BMI decreased significantly from baseline to post-program (−0.5 or 1.5% of baseline BMI, *P* < 0.001) and from post-program to follow-up (−1.3 or 3.7% of baseline BMI, *P* < 0.001) which is approximately 3 kilograms. After adjusting follow-up body weight for potential self-report bias, follow-up BMI (mean = 32.2, ±SD = 5.8) was still significantly lower than measured post-program BMI (−0.7, *P* < 0.001). After recalculating follow-up BMI using measured rather than self-reported height, follow-up BMI (mean = 31.8; ±SD = 5.7) was also still significantly lower than post-program BMI (−1.1, *P* < 0.001).

There were no significant differences observed in BMI change during the program period among the three baseline weight status groups. However, a significant difference was found in follow-up BMI change among weight status groups; overweight participants lost less weight than did obese and very obese participants (*P* < 0.01), Table 3.

Seventy-four percent of SWHH participants (*n* = 114) maintained their post-program BMI (or lost additional body weight) during the follow-up period, with a mean weight loss of −5.16 kilograms (±SD = 5.72) while 26% gained weight (*n* = 40), with a mean weight gain of +3.08 kilograms (±SD = 3.77) (*P* < 0.001); data not shown. These data also show that 72% of those who decreased their BMI during the program maintained it post-program, and similarly 79% of those who lost no body weight during the program also lost weight or maintained their body weight post-program, a difference that is not significant (chi-square = 0.43; data not shown).

There were statistically significant correlations between self-efficacy for healthy eating and follow-up BMI change (*r* = −0.17, *P* = 0.04) and between friends encouragement of healthy eating and follow-up BMI change (*r*_*pb*_ = −0.20, *P* = 0.02), Table 4. There were no associations between self-efficacy and social support for physical activity and follow-up BMI change.

## 4. Discussion

This study aimed to understand BMI maintenance among former SWHH program participants approximately three years later as well as to examine relationships with BMI maintenance and social support and self-efficacy. Overall, SWHH participants maintained their weight loss and, in some cases, lost additional weight over the three years after the program ended. On average, participants further reduced their weight by more than three percent of baseline BMI, or about three kilograms. According to results from a national sample of adults who successfully maintained weight loss for at least one year, a little over half of the sample reported they had lost weight through formal programs and the rest lost weight on their own [32], but the data in this area are very limited.

On average, SWHH participants with higher baseline BMI (obese and very obese categories) had significantly more BMI reduction in the follow-up period than did participants with lower baseline BMI (overweight category), despite equivalent weight loss in the program period. This is possibly due to response bias. One prior review found that successful weight maintenance was associated with more initial weight loss [12], but these data suggest otherwise.

Self-efficacy for healthy eating was associated with successful long-term BMI maintenance, similar to other studies [33]. Social support for healthy eating was likewise associated with long-term BMI maintenance. Results of other studies that examine social support and weight loss maintenance are inconsistent, although there is some evidence that compliments and active participation may be helpful while verbal instruction and encouragement may be perceived negatively [18]. To help elucidate the role of social support in weight maintenance, future studies may use mixed methods in which type of support is examined and qualitative data are collected to determine how different types of support are perceived. Contrary to other studies [22, 34], self-efficacy and social support for physical activity were unrelated to maintenance; this is potentially the result of differences in study populations, intervention strategies, and measurement of self-efficacy. Maintenance of physical activity and/or dietary behavior change is often not reported in weight maintenance studies, according to a systematic review of the evidence [35]. One review found that successful weight maintenance was associated with behaviors such as having a physically active lifestyle, regular meal rhythm, control of over-eating, and self-monitoring of behaviors [12].

There are some limitations of this study worth noting. Height and body weight at baseline, and body weight post-program were measured, whereas follow-up data collection relied on self-reported height and weight. Relative to objective measures, self-reported height is generally overestimated and body weight is underestimated [30]. There is some evidence to suggest that reporting bias may have contributed to declines in BMI observed during the follow-up period. Higher baseline BMI was related to larger decreases in BMI during the follow-up period but was unrelated to BMI change during the program period. If larger decreases in BMI among individuals with higher body weight were due to the relatively larger amount of weight to lose, we would expect this relationship to hold true during both time periods. Neither of our two approaches to adjusting BMI for self-report bias influenced our findings or their statistical significance, suggesting that bias did not account for the entire observed difference. Additionally, these data only represent one follow-up time point; it will be important to collect additional data in the future to understand long-term maintenance and possible weight cycling. It is possible that SWHH follow-up study responders were more successful with weight maintenance than nonresponders. Nevertheless, even if responders maintained their weight more often than nonresponders, responder weight maintenance rates are similar to, or higher than, what other studies have reported [36].

## 5. Conclusion

These findings support the durability of weight loss following participation in a relatively short-term intervention. Self-efficacy for healthy eating and social support for healthy eating were related to successful BMI maintenance.

## Figures and Tables

**Table 1:** Baseline characteristics and post-program BMI change by SWFUS response.

Characteristics at baseline	No follow-up response^a^ (*n* = 446), mean (±SD), or %	SWFUS respondents^b^ (*n* = 154), mean (±SD), or %	*P* value
*Mean age (±SD)*	60.1 (10.0)	58.9 (8.4)	0.19
*Race/ethnicity, %*			0.09
White, non-Hispanic	92.6 (0.3)	88.1 (0.3)	
*Education, %*			0.81
High school or less	25.4	23.8	
Some college	35.5	37.3	
Bachelor's degree	20.2	17.5	
Graduate training	18.9	21.4	
*Employed, yes, %*	49.6	51.4	0.72
*Household income level, %*			0.56
<$50,000	54.9	49.6	
$50,000–74,999	22.8	22.1	
$75,000–100,000	13.3	16.0	
>$100,000	9.0	12.2	
*Married, yes, %*	67.7	77.7	0.03^*∗*^
*Mean BMI (±SD), baseline*	33.6 (6.1)	33.4 (6.1)	0.74
*Mean BMI change (±SD), baseline to post-program*	−0.50 (0.9)	−0.50 (0.9)	0.99

^a^Sample size varied from 397 to 446 due to missing data.

^b^Sample size varied from 126 to 154 due to missing data.

Differences across follow-up response were tested using Chi-square analyses and *t*-tests. Boldface indicates statistical significance (^*∗*^*P* < 0.05).

BMI, body mass index; SWFUS, StrongWomen Follow-Up Study.

**Table 2:** Mean BMI at baseline, post-program, and follow-up (*n* = 154).

	Baseline	Post-program	Follow-up^a^
	Mean (±SD)	Mean (±SD)	Mean (±SD)
BMI	33.4 (6.1)	**32.9**(6.0)^*∗∗∗*^	**31.6**(5.8)^*∗∗∗*^
% of baseline BMI	100.0 (0.0)	**98.5**(2.7)^*∗∗∗*^	**94.8**(7.8)^*∗∗∗*^

^a^Mean self-reported unadjusted follow-up BMI.

Boldface indicates statistical significance (^*∗∗∗*^*P* < 0.001) for paired *t*-tests between this measure and the prior column.

BMI, body mass index.

**Table 3:** Mean program and follow-up BMI change by weight status groups (*n* = 154).

	Weight status		
	Overweight (*a*) (*n* = 50)	Obese (*b*) (*n* = 53)	Very obese (*c*) (*n* = 51)
	Mean (±SD)	Mean (±SD)	Mean (±SD)
Program BMI change	−0.43 (0.79)	−0.43 (0.89)	−0.64 (0.97)
Program BMI change as % of baseline BMI	−1.56 (2.93)	−1.35 (2.74)	−1.59 (2.42)
Follow-up BMI change	**−0.30 **(1.97)^*b*,*c*^	**−1.51 **(2.27)^*a*^	**−2.24 **(3.01)^*a∗∗∗*^
Follow-up BMI change as % of baseline BMI	**−1.07 **(7.24)^*b*,*c*^	**−4.52 **(6.94)^*a*^	**−5.55 **(7.47)^*a∗∗*^

Note: *a*, *b*, and *c* indicate which means are significantly different from each other via post hoc tests (Tukey).

Boldface indicates statistically significant *F*-test at ^*∗∗*^*P* < 0.01 and ^*∗∗∗*^*P* < 0.001.

BMI, body mass index.

**Table 4:** Correlation between self-efficacy and social support for healthy eating and physical activity and BMI change.

	Follow-up BMI change (*n* = 153)	Follow-up BMI change as % of baseline BMI
Self-efficacy and social support		
*Healthy eating*		
Self-efficacy for healthy eating	**−**0.17^*∗*^	**−**0.18^*∗*^
Social support for healthy eating		
Family encourages	**−**0.05	**−**0.04
Family discourages	**−**0.04	**−**0.03
Friends encourage	**−**0.20^*∗*^	**−**0.19^*∗*^
Friends discourage	**−**0.10	**−**0.03
*Physical activity*		
Self-efficacy for physical activity	**−**0.07	**−**0.08
Social support for physical activity		
Family participates	+0.05	+0.04
Friends participate	+0.06	+0.07

Boldface indicates statistical significance (^*∗*^*P* < 0.05).

BMI, body mass index.
